# Health information, what do people search and where? a cross-sectional online survey study in the adult Swiss population

**DOI:** 10.1371/journal.pone.0312120

**Published:** 2024-10-11

**Authors:** Laura Diaz Hernandez, Roland Fischer, Andreas Zeller

**Affiliations:** Centre for Primary Health Care, University of Basel, Basel, Switzerland; University Hospital Cologne: Uniklinik Koln, GERMANY

## Abstract

Health promotion and disease prevention are crucial for improving public health and alleviating the burden of illness in the population. This study aimed to investigate, the sources of health information most used and trusted, and the health topics most searched, by means of a nationwide cross-sectional online survey of a representative sample of the adult Swiss general population. Overall, complete surveys of 2020 participants were analysed (mean age 47 years old, 51% male, and matching the Swiss population regarding age, sex, and language-speaking region). Sources’ use were calculated with descriptive statistics per sex and age groups. The most frequently used sources were general practitioners (min: 46% to max: 73%), government websites (40% to 55%), family and friends (37% to 63%), pharmacy (33% to 46%), and television (21% to 57%). The most trusted sources were specialised physicians (94% to 98%) general practitioners (90% to 96%), and pharmacies (81% to 89%). Based on multivariable controlled regression, age (per five years increase) was associated with increased odds of using television (Odds Ratio (OR): 1.19, 95% Confidence Interval (CI): 1.12 to 1.21), print media (OR: 1.15, 95% CI: 1.10 to 1.19), radio (OR: 1.15, 95%CI: 1.10 to 1.20), and the general practitioner (OR: 1.11, 95% CI: 1.07 to 1.15), and decreased odds of using news websites (OR: 0.94, 95% CI:0.9 to 0.98), family and friends (OR: 0.93, 95% CI: 0.9 to 0.98), foreign authorities websites (OR: 0.91, 95%CI: 0.86 to 0.97), and social media (OR: 0.88, 95% CI: 0.84 to 0.92). Women were more likely to seek health information in the pharmacy (OR: 1.39, 95% CI: 1.15 to 1.68), specialised physicians (OR: 1.39, 95% CI:1.13 to 1.72), television (OR: 1.41, 95% CI: 1.16 to 1.72), and books (OR: 1.9, 95% CI: 1.44 to 2.5). The most searched health topics, based on the International Classification of Primary Care, 2^nd^ edition (ICPC2), were general and unspecified symptoms (20.9%) and musculoskeletal issues (19.4%). The use of these findings by policymakers and health care providers could potentially enhance the effectiveness of health-related education strategies, by aligning communication efforts with the populations’ preferences and content needs, and allocating resources where they are most commonly accessed and trusted, namely in the general practice.

## Introduction

### Background

Health promotion and disease prevention are essential for improving public health and alleviating the burden of illness in the population. They aim to reduce risk factors and increase healthy behaviours, like healthy lifestyles, vaccinations, early detection screenings, and timely interventions [1]. Equally crucial for effective prevention and health promotion is health literacy, which refers to the ability to access, understand, evaluate, and use health-related information. Higher health literacy enables individuals to make informed decisions about their health, and to adopt healthy behaviours leading to better health outcomes and public health [2]. Conversely, low health literacy is associated with poorer health outcomes and lower use of health care services [3]. In the digital age, health literacy also involves navigating online sources and assessing their reliability [4–6].

Myriad sources and communication formats provide health information, and thus convey the same knowledge in different ways, leading to different levels of comprehension and engagement. Most importantly different sources of information influence behaviour differently [7]. Therefore, it is relevant to assess which are the preferred sources used by the population to inform the best practices to tailor public health education, however considering that individuals encounter different sources of health-related information simultaneously, and thus there is an interlay of influences from all sources.

Primary healthcare providers, such as general practitioners (GPs), specialist physicians (e.g. cardiologists), and pharmacists, are the first point of contact for patients in the healthcare system and play a key role in managing various health conditions. A study in Switzerland showed that 16.4% of the population consulted their general practitioner within two months, while 8% engaged with a private care specialist, and 2% each visited emergency departments or pharmacies [8]. Trust in primary care is a key determinant of patient satisfaction, health outcomes, and adherence to medical advice. A study in the United States showed that a longer patient-physician relationship in primary care improved communication, knowledge, and trust [9]. In turn, trust associated with higher rates of preventive services, such as influenza vaccination, mammography, and eye examinations for diabetics [9]. However, another study in rural areas of the United States found no significant associations between physician continuity and use of recommended preventive services [10].

Family and friends are important sources of health-related information and support for individuals, particularly older adults [11]. They help to manage personal health information and shape health behaviours through trust and influence [12].

The internet and social media are important tools for health promotion and disease prevention. They offer a space for sharing experiences, seeking advice, and finding support, as well as providing health-related information. However, the quality and reliability of online information vary widely, and users need to evaluate the sources carefully.

Traditional media, including television (TV), radio, and newspapers have long been a trusted source of information for many people. These platforms often disseminate health-related information to the public, ranging from preventive measures to updates on public health crises. The trust in traditional media stems from the perception that these media outlets are regulated and adhere to professional standards, although their trust for health information has been declining over the past decade [13,14] while it has increased for interpersonal sources, like physicians [15].

Worldwide and in Switzerland, chronic diseases are mainly caused by alcohol, smoking, physical inactivity, and unhealthy diet. Switzerland aligns its efforts to prevent these issues with the World Health Organization (WHO) action plans both globally and in Europe [16]. However, other health issues also require attention, such as long COVID, mental health, cardiovascular disease, lower respiratory infections, diabetes, dementia, and population ageing [17].

### Objectives

Health issues vary in salience and trust among different demographic groups, which implies the need for tailored health information to reach diverse populations effectively. To produce such information, it is necessary to assess the population’s actual behaviour to learn about health. Thus, this study aims to investigate the sources of information, the level of trust, and the health topics that a representative sample of the Swiss population used to learn about health promotion and disease prevention in the previous 12 months.

## Methods

### Study design

A nationwide cross-sectional online survey conducted in a representative sample of the adult Swiss general population.

### Setting, participants’ selection and data acquisition

In November 2022 (from 30.10.2022 to 12.11.2022) the LINK Institute, Zürich, Switzerland (https://www.link.ch/) invited 14’501 adult persons to participate in the web-based survey in order to collect 2000 complete and valid questionnaires. The participants were selected from an online panel of 115’000 active recruited members, based on quotas (e.g., quota 1 = German- speaking region, male, between 18 and 29 years old). No contacts were excluded, and Rim weighting [18] was used to adjust for quotas overflow and to ensure that the drawn sample was representative of the Swiss population, according to age, sex, and language region [19].

### Survey and variables

The survey utilised a fully structured questionnaire consisting of both semi-open and closed questions, with an average completion time of approximately five minutes. The survey was developed in our Institute considering the most common different sources of information to learn about health promotion and disease prevention. It was internally revised for clarity and comprehension by the authors of the study and by the Link Institute staff responsible for the project. After approval, the Link Institute translated it into German, French and Italian.

Participants reported their actual behaviours regarding the acquisition of health-related information. They had a randomized list of media and interpersonal information sources, and for each item they had to indicate if they had used it or not (binary response yes/no) during the previous 12 months.

They also indicated the level of trust they attributed to each source, on a four-point scale, spanning from one (no trust at all) to four (a significant degree of trust). The specific media sources were Tv, radio, print (magazines and newspapers), social media (Facebook, X (Twitter), Instagram, TikTok, Reddit, WhatsApp/Telegram groups), Swiss federal websites (i.e. Ministry of Health website), foreign authorities websites (i.e. US Food and Drug Administration), news websites, books, and scientific journals. The specific interpersonal sources included general practitioner (GP), specialized physician, pharmacy, health insurance, family and friends, and community organizations (i.e. clubs). Furthermore, they wrote which specific health topics they had sought information on, excluding COVID-19.

The survey also asked if participants were registered with a (GP), the frequency of preventive healthcare discussions with their GP, and a self-assessment of their own health status on a four-point scale (ranging from one, poor health, to four, very good). Essential demographic information was collected including: sex, age (and age group 18–29, 30–44, 45–59, 60+ years old), language-speaking-region (German, French, Italian), residential environment (urban, rural), household size (one to two people, three or more people), education level (primary-medium education, higher education), household income (< 6000 Swiss francs (CHF,) 6001–10000 CHF > 10000 CHF, No answer), profession (owner/executive office, employee, learning, at home, not working), and, employment status (yes part-/full-time, no). Detailed survey questions are accessible in the (S1 Survey in S1 File).

### Analysis

The analysis was done on weighted data, with R software [20] and primarily with the R package "survey" [21]. Descriptive analysis was employed to examine the frequency of use of each information source and their associated trust rating as a binary response (no/low trust, responses 1 and 2 vs high trust, responses 3 and 4). Social media sources were merged into a “social media” category including YouTube, Facebook, X (Twitter), Instagram, TikTok, Reddit, and WhatsApp/Telegram groups.

Health topics were assessed with an open question. The responses to this open-ended question were categorised by the first author. Each answer was looked individually and assigned as many codes as necessary from the International Classification of Primary Care, 2^nd^ edition (ICPC2 [22]). ICPC2 is an accepted and commonly used coding system to record health problems and medical measures in primary care settings. It provides a standardised way to code information form primary care, facilitating data collection and analysis. Additional categories were created (not present in ICPC2), namely “none, “COVID”, “alternative remedies”, and “insurance/registry”. Although individuals were asked to exclude to report information seeking for COVID-19, a considerable number of participants mentioned searching for COVID-19. Therefore, we were obliged to add an additional category labelled “COVID-19”. Questions about alternative remedies and insurance issues are not covered by the ICPC2 coding system. When necessary particular cases were discussed with the last author until an agreement on the most appropriate classification category was found. The frequency of appearance of each health topic category was computed, in total, per age group, and per sex.

Multivariable logistic regression was used to assess the differential reported use of health-information sources per sex and age group controlling for language-speaking-region, residential environment, household size, education, household income, and employment status.

### Ethical statement

Guidance was sought from the Ethics Committee of Northwest and Central Switzerland (EKNZ), which advised that formal ethical approval was not required since the survey complies with the general ethical and scientific standards for research with humans (Project-ID: Req-2022-01203).

Participants gave written consent at the beginning of the online survey, and data was collected anonymously.

## Results

Out of 14’501 originally sent surveys, 2020 individuals (14%) returned complete questionnaires. The mean age of the weighted sample was 47 years, 51% of respondents were male, 72% were German-speaking, 24% French-speaking, and four percent Italian-speaking, matching the distribution of the Swiss population. Further details about the demographic characteristics of respondents are presented in Table 1.

**Table 1:** Demographic characteristics of survey participants.

	Variable	n (%)
total N		2020.0
Sex	male	1029.9 (51.0)
	female	990.1 (49.0)
Age (mean (SD))		47.09 (16.20)
Age group (%)	18–29 years	296.5 (14.7)
	30–44 years	631.6 (31.3)
	45–59 years	573.5 (28.4)
	60+ years	518.5 (25.7)
Language region (%)	German	1453.2 (71.9)
	French	481.2 (23.8)
	Italian	85.7 (4.2)
Residential environment (%)	Urban	1623.5 (80.4)
	Rural	396.5 (19.6)
Household size (%)	1–2 people	1022.9 (50.6)
	≥3	997.1 (49.4)
Education (%)	Primary—Medium education	1001.1 (50.2)
	Higher education	991.1 (49.8)
Income (%)	<6’000 CHF	510.4 (25.3)
	6’001–10’000 CHF	661.6 (32.8)
	>10’000 CHF	552.5 (27.4)
	No Answer	295.5 (14.6)
Employment status (%)	Yes (part-/fulltime)	1442.6 (71.4)
	No	577.4 (28.6)

CHF = Swiss francs, SD = Standard deviation.

Overall, 92% of participants (95% CI: 90.7–93.11, n = 1848) declared to be registered with a GP, and 41% (95% CI: 39.21–43.63, n = 836) regularly discussed prevention with their GP. In a scale to self-rate health status from one (poor) to four (very good) 32% of participants reported it as very good (95% CI 29.44–33.61, n = 636), 58% good (95% CI 56–60.44, n = 1176), 9% rather poor (95% CI 7.7–10.32, n = 181), 1% poor (95% CI 0.85–1.94, n = 27). There were no significant differences (p = 0.277) per sex.

### Acquisition of health-related information: Sources’ use and trust per sex and age group

**Fig 1:**
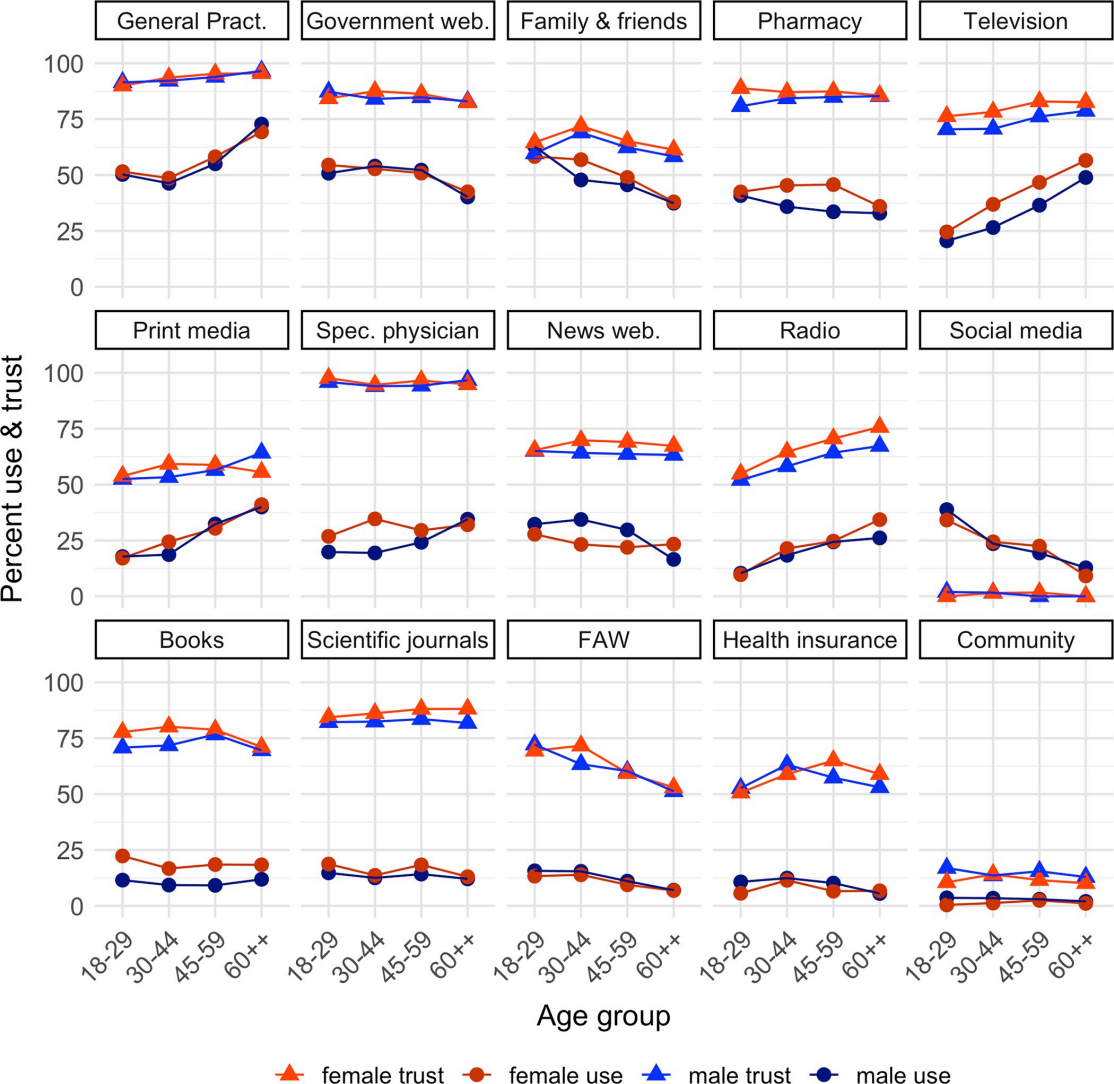
Use and trust of health information sources per age and sex. web. = websites, Spec. physician = specialised physician, FAW = Foreign Authority Website.

In the context of the past 12 months, the prevailing sources of health-related information most accessed by participants were the GP, government websites (e.g., Ministry of Health website), family and friends, pharmacy, and television. Fig 1 visually portrays the use percentages of all studied sources, stratified by both sex and age group.

Fig 1 also shows the degree of trust attributed to each source of information. The most trusted sources were the specialised physician, the GP, the pharmacy, and government websites.

**Fig 2:**
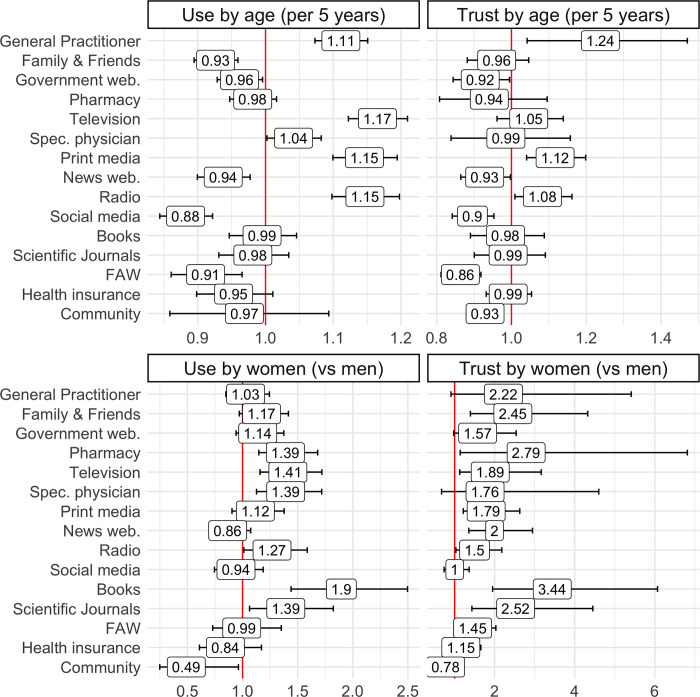
Association between age and sex and the use and trust of sources of health information (presented as ORs). Multivariable regressions modeling the association between age and sex, and the use and trust of each health information source studied, controlling for language region, residential environment, household size, education, income, and employment status. Regressions assessing the use of sources used 1997 observations, and regressions assessing the trust of sources used from 1210 observations (social media) to 1963 observations (general practitioner). Results are presented as Odds Ratios (OR) inside the squares. web. = websites, Spec. physician = specialised physician, FAW = Foreign Authority Website.

Multivariable logistic regression controlling for demographic variables (language speaking region, residential environment, household size, education, income, and employment status), indicated the differential use and trust of each information source per sex and age (five-year increments). Results are shown in Fig 2.

### Health-related topics searched

All ICPC2 codes except “Z- Social problems” were present in the respondents’ answers. Table 2 displays the frequency of health topics per category overall, and per sex, and age group.

**Table 2:** Frequency of health topics searched per sex and age group.

ICPC2 category and other	Total n (%)	Men 18-29y	Women 18-29y	Men 30-44y	Women 30-44y	Men 45-59y	Women 45-59y	Men 60+y	Women 60+y	p
	2020	111	167	362	290	273	296	283	238	
A) General and Unspecified	422 (20.9)	29 (26.1)	39 (23.4)	80 (22.1)	85 (29.3)	51 (18.7)	62 (20.9)	37 (13.1)	39 (16.4)	<0.001
B) Blood, Blood Forming Organs and Immune Mechanism	40 (2.0)	2 (1.8)	4 (2.4)	4 (1.1)	8 (2.8)	3 (1.1)	12 (4.1)	3 (1.1)	4 (1.7)	0.118
D) Digestive	187 (9.3)	7 (6.3)	16 (9.6)	32 (8.8)	19 (6.6)	25 (9.2)	33 (11.1)	28 (9.9)	27 (11.3)	0.493
F) Eye	45 (2.2)	4 (3.6)	3 (1.8)	7 (1.9)	6 (2.1)	4 (1.5)	4 (1.4)	12 (4.2)	5 (2.1)	0.304
H) Ear	35 (1.7)	0 (0.0)	1 (0.6)	4 (1.1)	9 (3.1)	8 (2.9)	5 (1.7)	6 (2.1)	2 (0.8)	0.142
K) Cardiovascular	174 (8.6)	8 (7.2)	7 (4.2)	21 (5.8)	19 (6.6)	23 (8.4)	27 (9.1)	48 (17.0)	21 (8.8)	<0.001
L) Musculoskeletal	391 (19.4)	16 (14.4)	19 (11.4)	43 (11.9)	45 (15.5)	42 (15.4)	80 (27.0)	62 (21.9)	84 (35.3)	<0.001
N) Neurological	129 (6.4)	7 (6.3)	18 (10.8)	20 (5.5)	21 (7.2)	8 (2.9)	28 (9.5)	12 (4.2)	15 (6.3)	0.010
P) Pyschological	144 (7.1)	5 (4.5)	12 (7.2)	25 (6.9)	29 (10.0)	13 (4.8)	27 (9.1)	17 (6.0)	16 (6.7)	0.220
R) Respiratory	206 (10.2)	15 (13.5)	16 (9.6)	52 (14.4)	32 (11.0)	24 (8.8)	31 (10.5)	25 (8.8)	11 (4.6)	0.013
S) Skin	191 (9.5)	11 (9.9)	16 (9.6)	37 (10.2)	24 (8.3)	19 (7.0)	31 (10.5)	32 (11.3)	21 (8.8)	0.740
T) Endocrine / Metabolic and Nutritional	212 (10.5)	9 (8.1)	16 (9.6)	31 (8.6)	42 (14.5)	26 (9.5)	35 (11.8)	27 (9.5)	26 (10.9)	0.305
U) Urological	52 (2.6)	0 (0.0)	7 (4.2)	4 (1.1)	11 (3.8)	6 (2.2)	4 (1.4)	9 (3.2)	11 (4.6)	0.027
W) Pregnancy, Childbearing, Family Planning	39 (1.9)	1 (0.9)	7 (4.2)	7 (1.9)	22 (7.6)	0 (0.0)	2 (0.7)	0 (0.0)	0 (0.0)	<0.001
X) Female Genital	83 (4.1)	2 (1.8)	20 (12.0)	7 (1.9)	23 (7.9)	0 (0.0)	26 (8.8)	1 (0.4)	4 (1.7)	<0.001
Y) Male Genital	60 (3.0)	2 (1.8)	5 (3.0)	9 (2.5)	7 (2.4)	10 (3.7)	3 (1.0)	24 (8.5)	0 (0.0)	<0.001
None	356 (17.6)	22 (19.8)	24 (14.4)	82 (22.7)	33 (11.4)	67 (24.5)	36 (12.2)	57 (20.1)	35 (14.7)	<0.001
Alternative remedies	24 (1.2)	0 (0.0)	2 (1.2)	1 (0.3)	6 (2.1)	1 (0.4)	8 (2.7)	3 (1.1)	3 (1.3)	0.067
COVID-19	83 (4.1)	4 (3.6)	3 (1.8)	14 (3.9)	9 (3.1)	17 (6.2)	14 (4.7)	12 (4.2)	10 (4.2)	0.477
Insurance/registers	10 (0.5)	0 (0.0)	1 (0.6)	1 (0.3)	1 (0.3)	5 (1.8)	1 (0.3)	1 (0.4)	0 (0.0)	0.088

Frequency of health topics reported in total and separated per sex and age group. p values indicate significant differences as computed with chi-square test.

We created a summary table accessible in the (S2 Summary in S1 File), including the most searched topics per sex and age group, along with the corresponding most used and trusted sources in the same population segments.

## Discussion

This study explored the sources used by a representative sample of the adult Swiss population to learn about health-related topics, along the trustworthiness of each source and the specific health topics checked. The results showed that GPs, family and friends, and government websites were the most common sources of information. GPs, particularly in elderly, specialised physicians, and pharmacy staff were the most trusted source of information on medical issues. As to expect, younger generations were more likely to resort to family and friends, and online media sources like news media websites, social media, and foreign authorities’ websites, whereas older generations were associated with a higher likelihood of contacting the GP, and checking traditional media sources like TV, print media (magazines and newspapers), and radio. The most searched health topics, based on ICPC2, were general and unspecified symptoms and musculoskeletal issues.

Health care providers were the most used and trusted sources. More than half of respondents reported to consult their GP, with a higher likelihood of consultation and trust with increasing age.

These results are consistent with our previous studies on the ecology of health care in Switzerland, where we showed that the main first medical advice comes from the GP [8], even during the COVID-19 pandemic [23]. Previous research has shown that older individuals are more likely to undergo check-ups and to seek health information than younger ones [24], and patients over the age of 64 living alone visit their GP more frequently [25]. Women were more likely to consult a specialised physician than men, maybe due to women regularly having gynecological examinations. In addition, women are more likely than men to seek advice from their health care professional and attend education sessions [24]. We hypothesise that the overall relative lower use of specialized physicians vs GPs, even though both groups were highly trusted, might be due to the Swiss health care structure, where GPs act as a gate keeping system for a majority of patients and the presumably longer waiting lists of the specialised physicians. Likewise, the pharmacy was another used and trusted source, contacted by half of respondents. A review study in Canada suggested that pharmacists have a major role in primary care due to their accessibility, as they see patients 1.5 to 10 times more often than GPs do. According to previous research, we found women more likely to use the pharmacy, as men generally use less community pharmacies and health care services [26]. Evidence suggests that factors influencing patients’ trust in GPs include older age, marital status, higher education, urban residence, better health, and having a GP at all [27]. These factors might enhance and sustain trust, which in turn can improve the patient-physician relationship in primary care and health outcomes.

Family and friends were reported by almost half of the sample and more likely by the younger generations. The considerable reliance on informal personal connections for health-related information underscores our inherent nature as social beings. As suggested by a review on the implications of social connections, our bonds with others serve as a significant protective factor against morbidity and mortality [28]. This highlights the vital role of family and friends not only as pillars of emotional support but also as valuable resources for health-related information [28]. Furthermore, a scoping review about conceptual models of the health-promoting family, highlights the family as a key setting for health promotion, as it influences health behaviours and serves the purpose of health education [29].

Half of the respondents also sought health information from government websites, which were highly trusted. Correspondingly, other studies have shown that health care professionals, including government communications, are the most trusted sources of information and that trust is associated with the adoption of healthy behaviour [30]. However, online sources are becoming more popular, in particular in the United States, where many patients use online sources before consulting their doctors (even though they list their physicians as their preferred source of information). This highlights an important change in how patients consume health related information, using the internet before seeing a physician [31].

Respondents rarely used or trusted online sources (news websites, foreign authorities’ websites, and social media) to search health information, with the exception of younger individuals, according with previous literature [32]. Internet media do not generate trust, and this skepticism seems to be due to the lack of stringent fact-checking [33], and seems to vary according with education and health literacy level. A study showed that higher education and income were linked to higher trust in the internet, whereas there was a tendency for individuals with lower health literacy to rely on non-professional sources (TV, social media) for health information [15].

Traditional mass media, (radio, TV, and print: magazines, newspapers) were used by less than half of our sample, but favoured with increasing age. Trust levels of these sources were considerable albeit lower than for health care providers. Our findings are consistent with previous studies that have reported a decline in trust in traditional mass media for health information over the past decade, while trust in personal sources, such as doctors, has remained high [15]. Interestingly, the type of content plays a role, as a study found that important news, as those related to public life, were seen as more credible in traditional media (newspapers) than in internet media. However, for soft news, as entertainment and non-related to vital interest, the medium did not matter in the level of trust. In addition, a meta-analysis showed that media campaign-generated interpersonal communication addressing health issues increased the odds of achieving campaign goals by 28%, but this effect was moderated by the health topic, the campaign goals, and the individuals involved [34].

To ensure the quality and reliability of the information disseminated, particularly for the communication strategies addressed to the younger age groups, internet media, and in particular social media needs monitoring for quality in health information, as recommended by a systematic review [35]. In addition, it is necessary to enhance the public’s ability to evaluate the quality and reliability of information sources [36]. According to a recent survey, nearly half of the Swiss population (49%) frequently experiences difficulties in dealing with health information, indicating a significant prevalence of low health literacy [37]. To address this issue (among others) the Swiss Federal Council launched a comprehensive health policy strategy, Health2030, which aims to promote health literacy through public information campaigns and improve the utilisation of health and disease-related information [38]. This integrated approach seeks to bridge the existing gap in health literacy, empower individuals with knowledge, and promote proactive health behaviours, ultimately contributing to a healthier and more informed population.

According to the Swiss Federal Office of Public Health’s 2023 Statistics, 85% of the population rates their health as either good or very good [39]. This finding is consistent with our sample evaluation, in which a notable 90% of participants reported feeling either good (58%) or very good (32%), suggesting that the self-perceived health status within the Swiss population is predominantly positive. Nonetheless, positive health perceptions do not negate the need for regular health information, thus identifying common health conditions in a population provides key insights into health-related needs and priorities. As health topics are classified according to its closest ICPC2 categories, the reader should keep in mind this broad perspective when interpreting the results. As expected, the most common topics belong to the category "General and Unspecified”, followed by the ICPC2 category “Musculoskeletal”. Important to mention is also the fact that a relevant proportion of participants was looking for information on psychological health issues.

Regarding to mental health, our findings indicate that the COVID-19 pandemic might have had a significant impact, particularly on younger generations [40]. According to a recent report, while a large portion of the population reports experiencing more positive than negative emotions, 14% display signs of psychological distress. Notably, young individuals aged 15 to 24 frequently suffer from mental health issues [41]. However, our analysis did not reveal significant differences in psychological topics among different age groups or sex. It is important to note that the “Psychological” category, based on ICPC2, encompasses a variety of aspects including feelings of depression, concerns about sexual preferences, stuttering, tics, tobacco use, dementia, hyperkinetic disorder, and more. The broad nature of the category could explain why our results did not show significant differences between age groups.

The European Commission Directorate-General for Research and Innovation identified high-burden under-researched medical conditions, referring to conditions that do not receive enough research funding compared to the funding produced by their burden. Among other conditions, they list musculoskeletal, skin and subcutaneous, digestive, and mental disorders [42]. The active search for these topics in our study and their inclusion in the aforementioned list, might reflect a genuine need for funding and research and subsequent dissemination of information to the general population to cover this need for evidence-based information, and therefore improve health outcomes and quality of life for those affected.

### Limitations and future directions

The cross-sectional online nature of this survey study has several inherent limitations. First, the data collected represents a specific point in time and does not account for changes over time. This restricts our ability to infer causality or temporal relationships between variables. Second, the reliance on self-reported data may introduce response bias, as participants might provide socially desirable responses or misinterpret questions. Third, despite efforts to ensure a diverse sample, the online nature of the survey may exclude individuals without internet access or digital literacy skills, potentially limiting the generalizability of our findings. Lastly, non-response bias could be present if those who chose to participate in the survey differ significantly from those who did not.

The recent COVID-19 pandemic may have influenced our results. Notably, 4% of participants reported searching for COVID-19, despite explicit instructions to exclude it. This instruction could have been both advantageous and disadvantageous. On one hand, it encouraged participants to consider other health-related topics of interest, potentially limiting responses solely focused on COVID-19 due to its prominence. On the other hand, the mention of COVID-19 in the question might have inadvertently prompted participants to think about it. This highlights the complexity of conducting research during a global pandemic and the potential influence of such significant events on participant responses.

Health literacy is associated with the trust placed in an information source, and in turn, trust is related to behavioural change [15]. Unfortunately, we did not measure health literacy in our sample and we suggest future studies to assess it to gain a more comprehensive understanding of health information-seeking behaviours to improve health education strategies.

Our study has some limitations. First, the topics were assessed with an open question, and primarily the first author coded the responses, discussing unclear cases with the last author. Second, some health topics reported were too unspecific to be assigned to an ICPC2 category. For example, for issues affecting the genital apparatus, if not specified whether it was for men or women, both categories (X and Y) were assigned. This approach ensures a comprehensive analysis despite the lack of specificity. Furthermore, using an internationally recognized classification system in general practice such as ICPC2 can enhance the clarity and credibility of the results. Further, it ensures that the findings align with established standards, and are comparable and usable for an international audience.

### Implications for health promotion and prevention

This study elucidates the sources used by the adult Swiss population to learn about health topics and evaluates the sources’ trustworthiness. It also identifies the most frequently searched health topics, indicating areas that need increased educational efforts. The use of these findings by policymakers could potentially enhance the effectiveness of their health-related campaigns. Tailoring communication channels and formats to align with source preferences, collaborating with healthcare providers to bolster patient education, and leveraging digital platforms for targeted outreach, could accomplish this. In terms of content development, aligning health content with community interests, would make it relevant to the population, and thus would contribute to optimize educational initiatives. Moreover, it is imperative to monitor regularly health-related information against scientific evidence, and intervene when necessary. Concurrently, promoting health literacy and critical thinking skills targeted at health-related information across various sources would be highly beneficial.

## Conclusions

This study provides highlights on health information seeking behaviour, trust in different information sources, and into the specific needs for health education of the Swiss population. The findings can inform health promotion efforts in Switzerland and beyond and help healthcare providers, policymakers, and researchers to develop content and development, research, and communication strategies. To maintain the high standard of medical care in Switzerland, we suggest allocation of resources where they are most commonly accessed and trusted, namely in general practice.

## Supporting information

S1 File (PDF)
