# Supplementary material for: Health information, what do people search and where? a cross-sectional online survey study in the adult Swiss population
Source: PLoS One. 2024 Oct 11;19(10):e0312120. doi: 10.1371/journal.pone.0312120 (PMC11469550; doi:10.1371/journal.pone.0312120)
Supplement: S1 File — (PDF) [file pone.0312120.s001.pdf]

Supporting Information

Article: Health information, what does people search and where? A cross-sectional online survey study in the adult Swiss population.

Laura Diaz Hernandez, Roland Fischer, Andreas Zeller

S1 Survey. Online survey in English

**Media sources**<br>**Which media health information sources have you used in the last 12 months to educate yourself about disease prevention?**
TV - health programs on television (e.g. Puls, Gesundheit Sprechstunde)
Radio
Print - magazines or newspapers
Youtube
Facebook
Twitter
Instagram
TikTok
Reddit
Whatsapp / Telegram Groups
Federal websites (e.g. BAG)
Websites of foreign authorities (e.g. FDA, CDC oder WHO)
Media websites (e.g. SRF, BBC oder CNN)
Books
Scientific journals
Other internet sources
None
**Interpersonal sources**<br>**Which of the following, interpersonal health information sources have you used in the last 12 months to educate yourself about disease prevention?**
General practioner
A specialised medical doctor
Pharmacy
Health insurance advice by phone
Family and friends or confidants
Community organisations (church, fraternities, associations, clubs)
Other health care providers
None
How much do you trust the information from each of these sources?<br>((not al all), 2, 3, 4 (a lot)
**On which health topics have you sought information in the las 12 months.** (excluding Covid-19)
**Are you registered with a general practitioner?** (Yes / No / I don't know)
**Do you regularly discuss preventive examinations with your general practitioner?**<br>Preventive examinations refer to tests and counselling sessions conducted by your doctor to prevent illness, including activities such as blood pressure control, assessing physical exercise levels, discussing smoking habits, reviewing vaccination status, and more." (Yes / No)
**How good would you rate your general health?** 1 (poor), 2,3,4 (very good)

S2 Summary. Summary of topics, sources and trust per age and sex groups.

WOMEN	Health topics most searched	Top used sources	Top trusted sources
**18-29y (n=167)**	General and unspecified (24%), Female genital (12%), Musculoskeletal (11%), Neurological (11%)	Family and friends (58%), Federal websites (55%), GP (51%)	Spec. physician (98%), GP (90%), Pharmacy (89%)
**30-44y (n=290)**	General and unspecified (29%), Musculoskeletal (16%), Endocrine/metabolic and nutritional (15%), Respirator (11%), Psycholgical (10%)	Family and friends (57%), Federal websites (53%), GP (49%)	Spec. physician (95%), GP (94%), Federal websites (88%)
**45-59y (n=296)**	Musculoskeletal (27%), General and unspecified (21%), Endocrine/metabolic and nutritional (12%), Digestive (11%), Respirator (11%), Skin (11%)	GP (58%), Federal websites (51%), Family and friends (49%)	Spec. physician (97%), GP (95%), Scientific journals (88%)
**60y (n=238)**	Musculoskeletal (35%), General and unspecified (16%), Digestive (11%), Endocrine/metabolic and nutritional (11%)	GP (69%), TV (57%), Federal websites (42%)	Spec. physician (95%), GP (95%), Scientific journals (88%)
**MEN**	**Health topics most searched**	**Top used sources**	**Top trusted sources**
**18-29y (n=111)**	General and unspecified (26%), Musculoskeletal (14%), Respiratory (14%)	Family and friends (63%), Federal websites (51%), GP (50%)	Spec. physician (6%), GP (91%), Federal websites (87%)
**30-44y (n=362)**	General and unspecified (22%), Respiratory (14%), Musculoskeletal (12%), Skin (10%)	Federal websites (54%), Family and friends (47%), GP (46%)	Spec. physician (94%), GP (92%), Pharmacy (84%)
**45-59y (n=273)**	General and unspecified (19%), Musculoskeletal (15%)	GP (55%), Federal websites (53%), Family and friends (46%)	Spec. physician (94%), GP (94), Pharmacy (85%)
**60y (n=283)**	Musculoskeletal (22%), Cardiovascular (17%), General and unspecified (13%), Respiratory (11%)	GP (73%), TV (49%), Federal websites (40%)	Spec. physician (97%), GP (97%), Pharmacy (85%)

S3. Regression analysis details

Regression results for use and trust of health information sources (age and sex)

source	OR	lCI	uCI	p	category
television	1.17	1.12	1.21	0.000	a. use age
radio	1.15	1.10	1.20	0.000	a. use age
print media	1.15	1.10	1.19	0.000	a. use age
social media	0.88	0.84	0.92	0.000	a. use age
government websites	0.96	0.93	1.00	0.028	a. use age
foreign authorities websites	0.91	0.86	0.97	0.002	a. use age
new websites	0.94	0.90	0.98	0.002	a. use age
books	0.99	0.95	1.05	0.841	a. use age
scientific journals	0.98	0.93	1.03	0.482	a. use age
general practitioner	1.11	1.07	1.15	0.000	a. use age
specialised physician	1.04	1.00	1.08	0.039	a. use age
pharmacy	0.98	0.95	1.02	0.289	a. use age
health insurance	0.95	0.90	1.01	0.109	a. use age
family and friends	0.93	0.89	0.96	0.000	a. use age
community	0.97	0.86	1.09	0.609	a. use age
television	1.05	0.96	1.14	0.295	b. trust age
radio	1.08	1.01	1.16	0.027	b. trust age
print media	1.12	1.04	1.20	0.002	b. trust age
social media	0.90	0.84	0.95	0.001	b. trust age
government websites	0.92	0.84	0.99	0.037	b. trust age
foreign authorities websites	0.86	0.81	0.92	0.000	b. trust age
new websites	0.93	0.86	1.00	0.042	b. trust age
books	0.98	0.89	1.09	0.754	b. trust age
scientific journals	0.99	0.90	1.09	0.855	b. trust age
general practitioner	1.24	1.04	1.47	0.015	b. trust age
specialised physician	0.99	0.84	1.16	0.856	b. trust age
pharmacy	0.94	0.81	1.10	0.432	b. trust age
health insurance	0.99	0.93	1.05	0.779	b. trust age
family and friends	0.96	0.88	1.05	0.356	b. trust age
community	0.93	0.89	0.97	0.000	b. trust age

television	1.41	1.16	1.72	0.001	c. use female
radio	1.27	1.01	1.59	0.039	c. use female
print media	1.12	0.90	1.38	0.307	c. use female
social media	0.94	0.74	1.19	0.606	c. use female
government websites	1.14	0.94	1.37	0.182	c. use female
foreign authorities websites	0.99	0.73	1.35	0.963	c. use female
new websites	0.86	0.70	1.07	0.189	c. use female
books	1.90	1.44	2.50	0.000	c. use female
scientific journals	1.39	1.06	1.82	0.016	c. use female
general practitioner	1.03	0.85	1.24	0.781	c. use female
specialised physician	1.39	1.13	1.72	0.002	c. use female
pharmacy	1.39	1.15	1.68	0.001	c. use female
health insurance	0.84	0.61	1.17	0.312	c. use female
family and friends	1.17	0.97	1.42	0.099	c. use female
community	0.49	0.25	0.96	0.039	c. use female
television	1.89	1.13	3.16	0.016	d. trust female
radio	1.50	1.03	2.18	0.034	d. trust female
print media	1.79	1.22	2.63	0.003	d. trust female
social media	1.00	0.74	1.36	0.989	d. trust female
government websites	1.57	0.98	2.54	0.062	d. trust female
foreign authorities websites	1.45	1.04	2.03	0.031	d. trust female
new websites	2.00	1.36	2.95	0.000	d. trust female
books	3.44	1.95	6.07	0.000	d. trust female
scientific journals	2.52	1.43	4.45	0.001	d. trust female
general practitioner	2.22	0.91	5.41	0.080	d. trust female
specialised physician	1.76	0.67	4.60	0.249	d. trust female
pharmacy	2.79	1.14	6.81	0.025	d. trust female
health insurance	1.15	0.81	1.66	0.433	d. trust female
family and friends	2.45	1.39	4.32	0.002	d. trust female
community	0.78	0.62	0.96	0.022	d. trust female
